# Assessment of *Babesia ovis* pathogenicity in goats: implications for transmission dynamics and host resistant

**DOI:** 10.3389/fcimb.2024.1480347

**Published:** 2024-11-12

**Authors:** Sezayi Ozubek, Mehmet Can Ulucesme, Carlos E. Suarez, Reginaldo G. Bastos, Munir Aktas

**Affiliations:** ^1^ Department of Parasitology, Faculty of Veterinary Medicine, University of Firat, Elazig, Türkiye; ^2^ Animal Disease Research Unit, United States Department of Agriculture (USDA) Agricultural Research Service, Pullman, WA, United States; ^3^ Department of Veterinary Microbiology and Pathology, College of Veterinary Medicine, Washington State University, Pullman, WA, United States

**Keywords:** *Babesia ovis*, experimental infection, goat, pathogenicity, *Rhipicephalus bursa*

## Abstract

*Babesia ovis*, commonly associated with ovine babesiosis, poses a significant threat to sheep health, often resulting in severe clinical manifestations and high mortality rates. However, the impact of *B. ovis* on goats has remained uncertain, prompting us to investigate its pathogenicity in caprine hosts. Experimental infections using *B. ovis*-infected blood inoculation and infected tick infestation, were conducted on spleen-intact (n=5) and splenectomized (n=5) goats. The experimental infection was performed using fresh blood obtained from a *B. ovis*-infected splenectomized sheep. One spleen-intact sheep served as a control for the experimental infection with *B. ovis*-infected *Rhipicephalus bursa* ticks. While all experimentally infected sheep (#501, #575) displayed severe clinical symptoms and high parasitemia, goats exhibited resistance, showing no significant clinical manifestations or sustained parasitemia. Notably, *B. ovis* was detected in two spleen-intact goats via nested PCR, prompting further investigation into their role as reservoirs for tick-borne transmission. These goats were then infested with *Babesia* spp.-free *R. bursa* larvae (0.1 gr) and adults (50 females and 50 males) for transstadial and transovarial transmission experiments respectively. Results indicated that chronically *B. ovis*-infected spleen-intact goats are not significant sources for maintaining the tick-borne transmission cycle of the parasite. These findings highlight the differential susceptibility of goats to *B. ovis* infection compared to sheep and their limited role as reservoirs for parasite transmission. Understanding the role of goats in *B. ovis* transmission and their resistance mechanisms can inform effective control measures and reduce economic losses in affected regions. Further research into caprine babesiosis and host immunological responses is essential to fully elucidate their possible role as reservoirs of the parasite, and underlying mechanisms of host susceptibility and parasite pathogenesis.

## Introduction

Goats, often referred to as the "poor man's cow," are renowned for their resilience and adaptability to harsh environments, making them indispensable assets for small-scale farmers worldwide (Kumar et al., 2006; Nair et al., 2021). In some countries, including Turkiye, goat breeding holds not only economic significance but also cultural importance, especially within nomadic communities (Alkan and Ugur, 2015). However, despite their hardiness, goats are susceptible to various infections, including babesiosis caused by *Babesia* spp., which poses a significant threat to livestock globally.

Babesiosis, the disease caused by *Babesia* spp., is known for its impact on livestock, particularly affecting sheep, cattle, and goats (Uilenberg, 2006; Schnittger et al., 2012, 2022; Ozubek et al., 2020). The economic consequences of babesiosis are profound, encompassing direct losses from animal morbidity and mortality, as well as indirect losses from reduced productivity and heightened veterinary expenses (Ozubek et al., 2020). Small ruminant babesiosis is commonly caused by *Babesia ovis*, *B. motasi*, and *B. crassa*. *Babesia taylori* and *B. foliata* were identified in India years ago, but information on these species remains limited (Schnittger et al., 2022). Over the past two decades, new species or genotypes of *Babesia* that affect sheep and goats have been identified, including *Babesia* sp. Xinjiang (Guan et al., 2009), *Babesia aktasi* (Ozubek et al., 2023a), and *B. motasi*-like species, such as *B. motasi* Lintanensis and *B. motasi* Hebeinensis (Wang et al., 2023).

*Babesia ovis* poses a significant threat to sheep, often causing severe clinical and hematological issues, with mortality rates reaching up to 50% (Yeruham et al., 1998a, 1998b; De Waal, 2000; Ceylan et al., 2021). The epidemiology of *B. ovis* is closely tied to the biology and ecology of *Rhipicephalus bursa* ticks (Yeruham et al., 1998a). *Rhipicephalus bursa* is a two-host species that can transmit *B. ovis* to hosts only during its adult stage (Erster et al., 2016). While the impact of *B. ovis* on sheep is well-documented, its effect on goats remains a subject of debate within the veterinary and scientific communities. Despite assertions in various review articles suggesting that *B. ovis* does not induce clinical infection in goats (Friedhoff, 1997; De Waal, 2000; Schnittger et al., 2022), recent studies have presented contrasting findings, indicating inflammatory responses and altered hematological parameters in naturally infected goats (Esmaeilnejad et al., 2012, 2020). Furthermore, molecular studies have detected *B. ovis* in goats across various geographical regions, including Turkiye (Aktaş et al., 2005; Aktas et al., 2007; Inci et al., 2010; Aktas and Özübek, 2017; Ulucesme et al., 2023), Tunisia (Rjeibi et al., 2014), Uganda (Tumwebaze et al., 2020), and Philippines (Galon et al., 2022), fueling discussions on the susceptibility of goats to the pathogen and its clinical implications (Schnittger et al., 2022).

Given the significance of goat farming for economic stability (Nair et al., 2021), clarifying the role of *B. ovis* in goat health is imperative. This study aimed to address the existing discrepancies in the literature by conducting controlled experimental infections in goats using both infected blood and infected ticks, mimicking natural transmission routes. Through comprehensive assessment, we determined whether *B. ovis* can indeed cause clinical babesiosis in goats. By elucidating the interactions between *B. ovis* and caprine hosts, in this study we provided new and valuable insights into the pathogenic potential of the parasite in goats. Such insights are crucial for developing effective management strategies to safeguard goat health and productivity, thereby ensuring the sustainability of small ruminant farming systems.

## Methods

### Ethics statement

This study was conducted in compliance with Turkish animal welfare regulations. All animal experiments received approval from the Firat University Animal Experiment Ethics Committee, under protocol number 2018/100.

### Selection of experimental animals and splenectomy

In this study, we used sheep and goats that were free of *Babesia*, *Anaplasma*, and *Theileria* infections. To select the experimental animals, we collected blood samples from apparently healthy sheep and goats aged 5 to 8 months. The samples were collected in both serum and EDTA tubes. We employed nested PCR (nPCR) with general primers to investigate the presence of these species. For *Anaplasma*, we used primers Ec9/Ec12A (Kawahara et al., 2006) and 16S8FE/B-GA1B (Bekker et al., 2002). For *Babesia* and *Theileria*, we used primers Nbab1F/Nbab1R (Oosthuizen et al., 2008) and RLBF2/RLBR2 (Georges et al., 2001), respectively. Six sheep and twelve goats were determined to be negative for these pathogens by nPCR. These animals were then transported to the Elazıg Veterinary Control Institute Directorate, where the experimental infections took place. They were kept in a tick-free environment and provided with feed and water ad libitum (Firat et al., 2024). Sheep (#501) and goat (#803, #862, #4K, #804, #SAN) underwent splenectomy at Firat University Veterinary Hospital and were subsequently housed in individual compartments to facilitate a two-week recovery period prior to the commencement of the experiment. The surgical procedure adhered to established protocols, ensuring standardized techniques for anesthesia and analgesia administration (Sevinc et al., 2007).

### Experimental infection by infected blood inoculation

**Figure 1:**
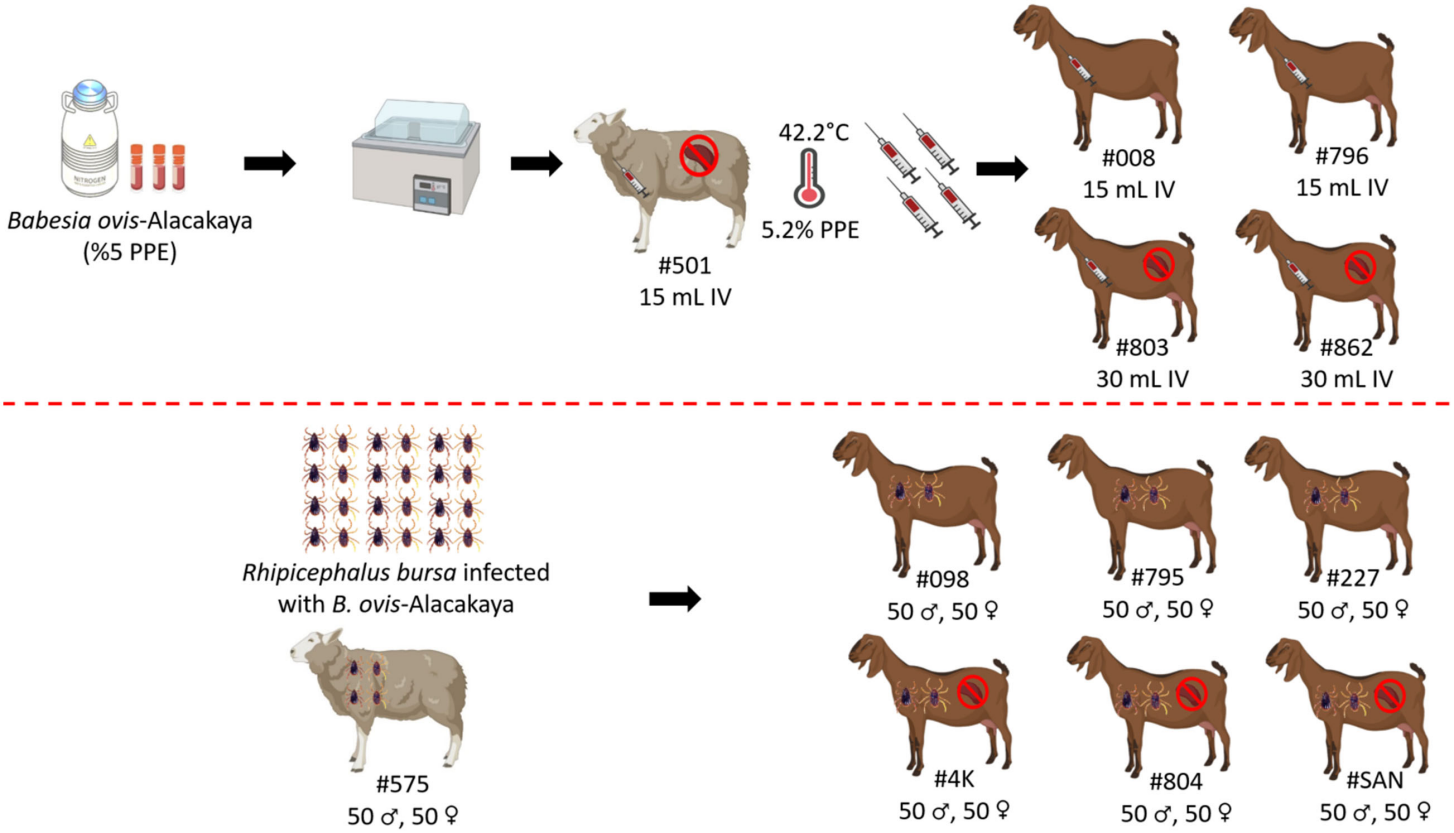
Schematic representation of sheep and goats used in experimental infection with infected blood and ticks with *B. ovis*-Alacakaya. Figure was created using Biorender.com.

The experimental infection was carried out as previously described by Guan et al. (2009) and Ozubek et al. (2023b). Briefly, the *Babesia ovis*/Alacakaya stabilate used in this study was originally obtained from a naturally infected sheep (Firat et al., 2024). To ensure the purity of the *B. ovis* stabilate and eliminate any potential contamination from other hemoparasites, such as *Theileria* or *Anaplasma*, the stabilate was subjected to transovarial passage through *R. bursa* ticks. Following this procedure, the stabilate was cryopreserved in a cryobank. Before experimental infection, the stabilate was reactivated by thawing and intravenously administered (15 mL) to a splenectomized sheep (#501). Once parasitemia reached 5.2%, 15 mL of fresh *B. ovis*-infected blood was intravenously administered to splenectomized goats (#803, #862), and 30 mL to spleen-intact goats (#008, #796) (Guan et al., 2009; Ozubek et al., 2023b; Firat et al., 2024). Blood samples were collected from the experimental donor animals daily for 60 days post-infection for analysis using microscopy and nested-PCR (nPCR) (**Figure 1**).

### Experimental infection by infected tick feeding

*Rhipicephalus bursa* ticks used in the experimental infections were obtained from a colony regularly maintained in our laboratory (Firat et al., 2024; Ulucesme et al., 2024). For the experimental infection, adult *R. bursa* ticks infected with *B. ovis* Alacakaya were used. The process of obtaining infected ticks is briefly described as follows: a splenectomized lamb experimentally infected with the *B. ovis* Alacakaya stabilate (Firat et al., 2024) was infested with 15 female and 10 male *Babesia* spp.-free *R. bursa* ticks. Engorged females were collected from the experimentally infected lamb and placed in an incubator (25 ± 1°C and 70 ± 10% relative humidity) to obtain infected larvae. Since *R. bursa* can only transmit *B. ovis* in the adult stage (Erster et al., 2016), the obtained larvae were used to infest a rabbit. Engorged nymphs were then collected from this rabbit. Finally, the engorged nymphs were placed in an incubator to obtain *B. ovis*-infected adult *R. bursa* ticks. As a control, one sheep (#575) was infested with 50 females and 50 males *R. bursa* adult ticks infected with *B. ovis*. Additionally, the same number of female and male *R. bursa* ticks were used to infest both splenectomized (#4K, #804, #SAN) and spleen-intact goats (#098, #795, #227). Following the experimental infection with the infected ticks, all animals (#575, #098, #795, #227, #4K, #804, #SAN) were monitored for clinical symptoms of ovine babesiosis, including increased body temperature, anemia, jaundice, and hemoglobinuria. Additionally, the animals were monitored for 60 days post-infection for *B. ovis* parasitemia using microscopy and nPCR methods (**Figure 1**).

### Tick acquisition and transmission feeding

Experiments on transstadial and transovarial transmission were conducted to determine if goats infected with *B. ovis* serve as a source of parasite transmission by *R. bursa* ticks. For the transstadial transmission experiment, infected goats (#098, #795) were each infested with 0.1 g *Babesia* spp.-free *R. bursa* larvae (Firat et al., 2024). Engorged nymphs were collected from these larvae-infested goats (#098, #795) and incubated at 25 ± 1°C and 70 ± 10% relative humidity (RH). After incubation, unfed adult ticks obtained from goats #098 and #795 were used to infest splenectomized sheep #K2 and #K4, respectively (50 females and 50 males per sheep).

**Figure 2:**
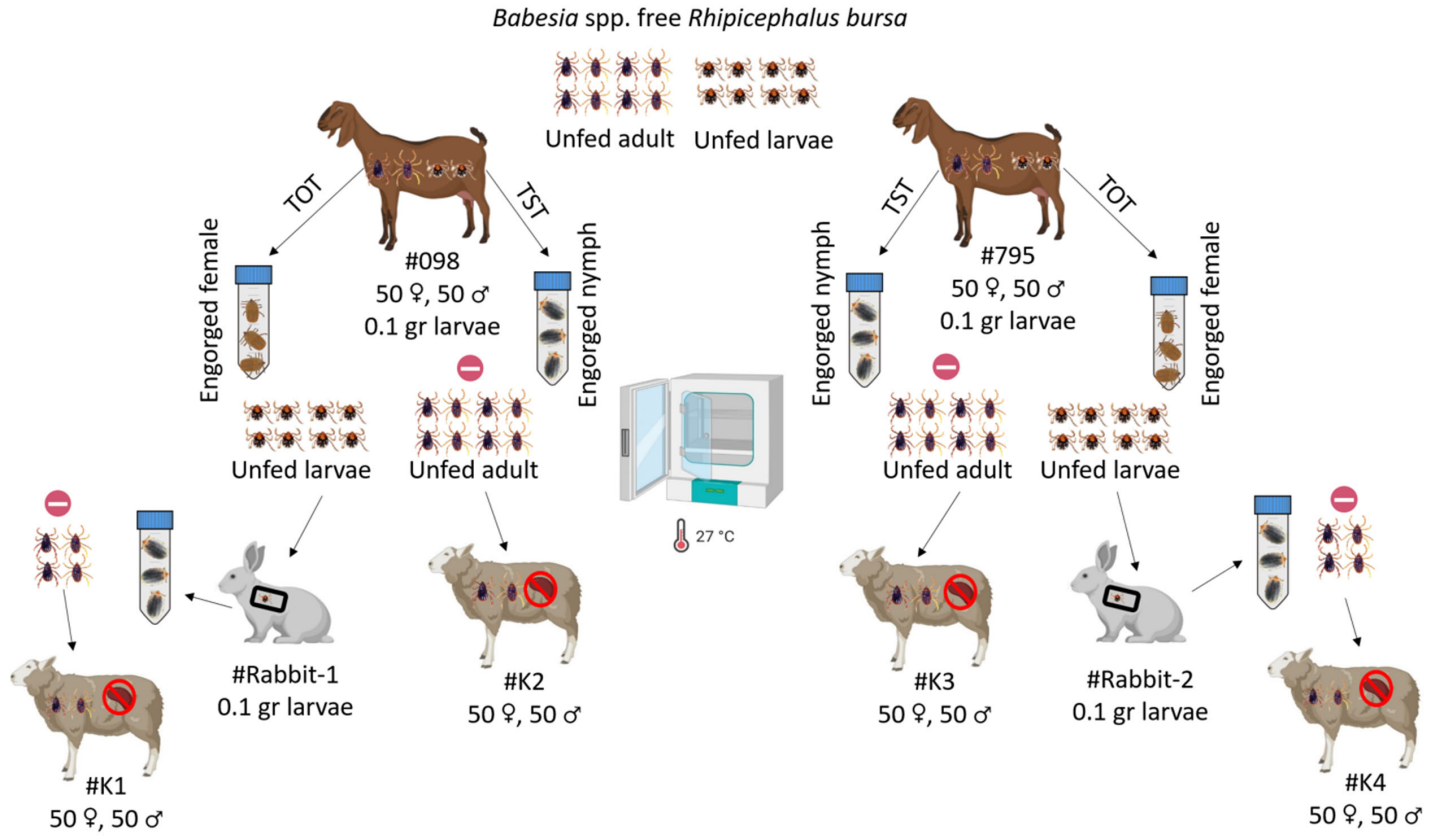
Schematic representation of transstadial (TST) and transovarial (TOT) transmission experiments using *Babesia* spp.-free *R. bursa* larvae and adults on goats infected with *B. ovis*. 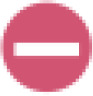: represent nPCR negative. Figure was created using Biorender.com.

For the transovarial transmission experiment, *Babesia* spp.-free adult *R. bursa* ticks were used to infest goats #098 and #795 (50 females and 50 males per goat). Engorged females were collected from each goat, placed in separate plastic containers, and incubated under the same conditions to obtain larvae. Larvae obtained from engorged females collected from goats #098 and #795 were used to infest #Rabbit-1 and #Rabbit-2, respectively. Engorged nymphs were collected from each rabbit and incubated under the same conditions to obtain unfed adult *R. bursa* ticks. Unfed adult ticks obtained from #Rabbit-1 and #Rabbit-2 were used to infest splenectomized sheep #K1 and #K4, respectively (**Figure 2**). Additionally, all splenectomized sheep (#K1, #K2, #K3, #K4) infested with adult *R. bursa* were monitored for 60 DPI for *B. ovis* using microscopy and nPCR methods.

Unfed adult ticks obtained from goats #098 and #795 and rabbits #Rabbit-1 and #Rabbit-2 were analyzed for the presence of *B. ovis* using nPCR. For this analysis, 60 unfed adult ticks (30 females and 30 males) from each goat and rabbit were divided into 12 pools (6 female pools and 6 male pools), with each pool containing 5 ticks (**Table 1**). These pools were stored in a -20°C for DNA isolation and subsequent nPCR analysis.

**Table 1:** Source and pooling of adult *R. bursa* from transstadial and transovarial transmission experiments.

Source of ticks	No of pools	No of female-male ticks pools	No. of ticks in each pool	No. of total ticks
#098	12	6-6	5	60
#795	12	6-6	5	60
#Rabbit-1	12	6-6	5	60
#Rabbit-2	12	6-6	5	60
#K1	100	50-50	1	100
#K2	100	50-50	1	100
#K3	100	50-50	1	100
#K4	100	50-50	1	100
Total	448	224-224	1 or 5	640

### Microscopic detection and nested PCR for *B. ovis*

For microscopic examination, thin blood smears were prepared from the ear tip of sheep, fixed in absolute methanol for 5 minutes, and stained with 10% Giemsa solution for 30 minutes. The slides were examined for intra-erythrocytic piroplasms using oil immersion at 100x magnification. Parasitemia, defined as the percentage of erythrocytes containing parasites, was assessed by counting infected erythrocytes in 20 randomly selected microscopic fields from the edge regions of the blood smear and compared with the total erythrocyte count. Mean PPE was calculated by dividing the number of parasitized erythrocytes by the total erythrocyte count. If no parasites were detected in 20 fields, the smears were recorded as negative for piroplasms (Sevinc et al., 2007; Ozubek and Aktas, 2017).

For the detection of *B. ovis* using nPCR, genomic DNA was extracted from 200 µL of EDTA-anticoagulated blood samples collected from sheep, goat, and *R. bursa* ticks used in experimental infections. The extraction was performed using the PureLink^TM^ Genomic DNA Mini Kit (Invitrogen Corporation, Carlsbad, USA) according to the manufacturer's instructions. The extracted DNA was then stored at −20°C until needed. The nPCR assay for identifying *B. ovis* DNA utilized two sets of primers: Nbab1F/Nbab1R (Oosthuizen et al., 2008) and BboF/BboR (Aktaş et al., 2005). For the detection of *R. bursa* DNA, PCR was conducted with the primers 16S + 1 and 16S – 1 (Black and Piesman, 1994), following established protocols.

## Results

### Absence of clinical signs of babesiosis in goats inoculated with fresh-*B. ovis* infected blood

**Figure 3:**
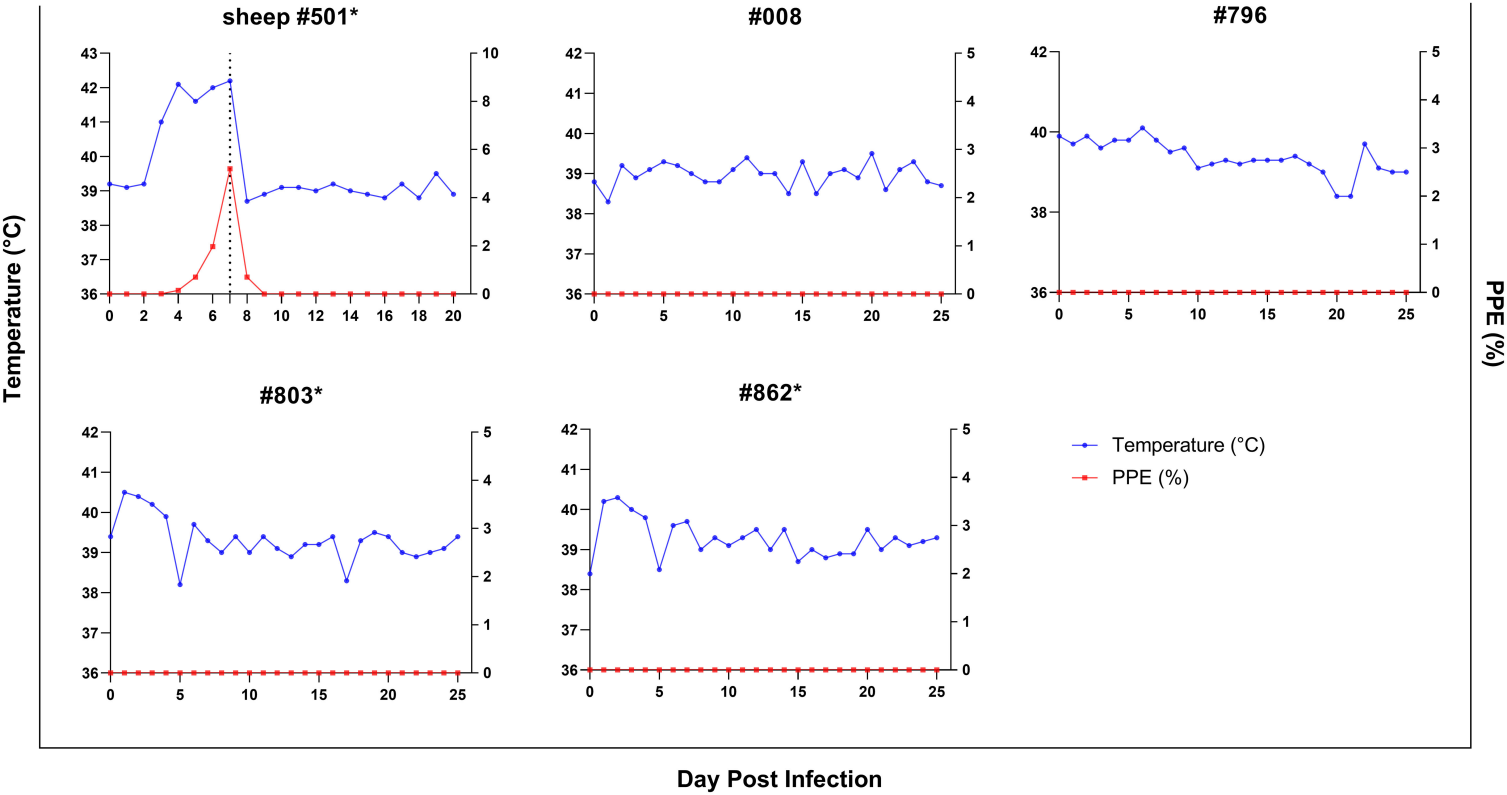
Parasitemia (PPE) (red lines) and body temperature (blue lines)in goats in splenectomized (*) and spleen-intact groups experimentally infected with *B. ovis*-infected fresh blood obtained from sheep #501. Day 7 in #501 shows the time 90 ml blood was drawn for the experimental infection of goats.

For investigating experimental infection of goats via direct blood inoculation, a *B. ovis* Alacakaya stabilate with a ~5% parasitemia percentage (PPE) stored in a cryobank was intravenously administered to a splenectomized donor sheep (#501) (**Figure 1**). Parasites were identified in the peripheral blood on the third day following infection. As the infection progressed, fever was observed, reaching peak temperatures between 41.0°C and 42.2°C, corresponding to an increase in parasitemia. On day 7, microscopic examination of peripheral blood smears revealed a PPE level of 5.2%. For the experimental infection of goats, 90 ml of the sheep infected blood was collected (**Figure 3**). At this point, the sheep exhibited severe clinical symptoms of babesiosis, including hemoglobinuria, jaundice, reduced appetite, and lethargy. Following blood collection, the sheep received treatment with imidocarb dipropionate (1.2 mg/kg). After treatment, the fever decreased to 38.7°C, and parasites were not detected in circulation upon microscopical examination by day 9 post-infection (DPI).

**Figure 4:**
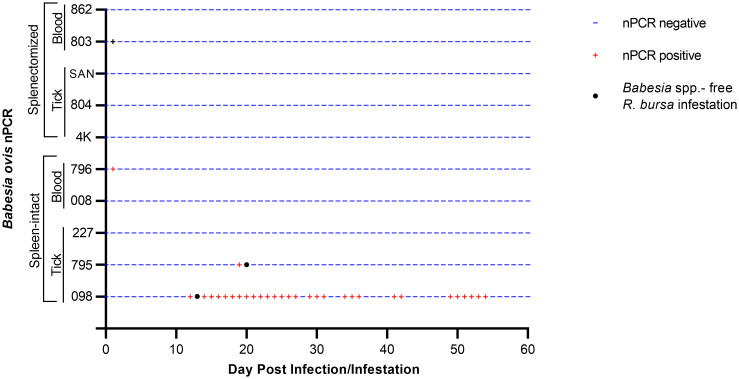
Monitoring of the presence of *B. bovis* DNA by nPCR in splenectomized and spleen-intact goats following a 60-day period of experimental.

Splenectomized goats (#803, #862) and spleen-intact goats (#008, #796) were infected with fresh-*B. ovis* infected blood from donor sheep #501 (**Figure 1**). The presence of *B. ovis* was monitored using microscopy and nPCR for the first 25 days, and only by nPCR from days 26 to 60. During this period, *B. ovis* remained undetected by any of these methods. However, *B. ovis* was identified by nPCR in goats #796 and #803 on day 1 post-infection (**Figure 3** and **Figure 4**). Additionally, no symptoms, such as fever, anemia, or jaundice, were observed in any of the goats throughout the experiment.

**Figure 5:**
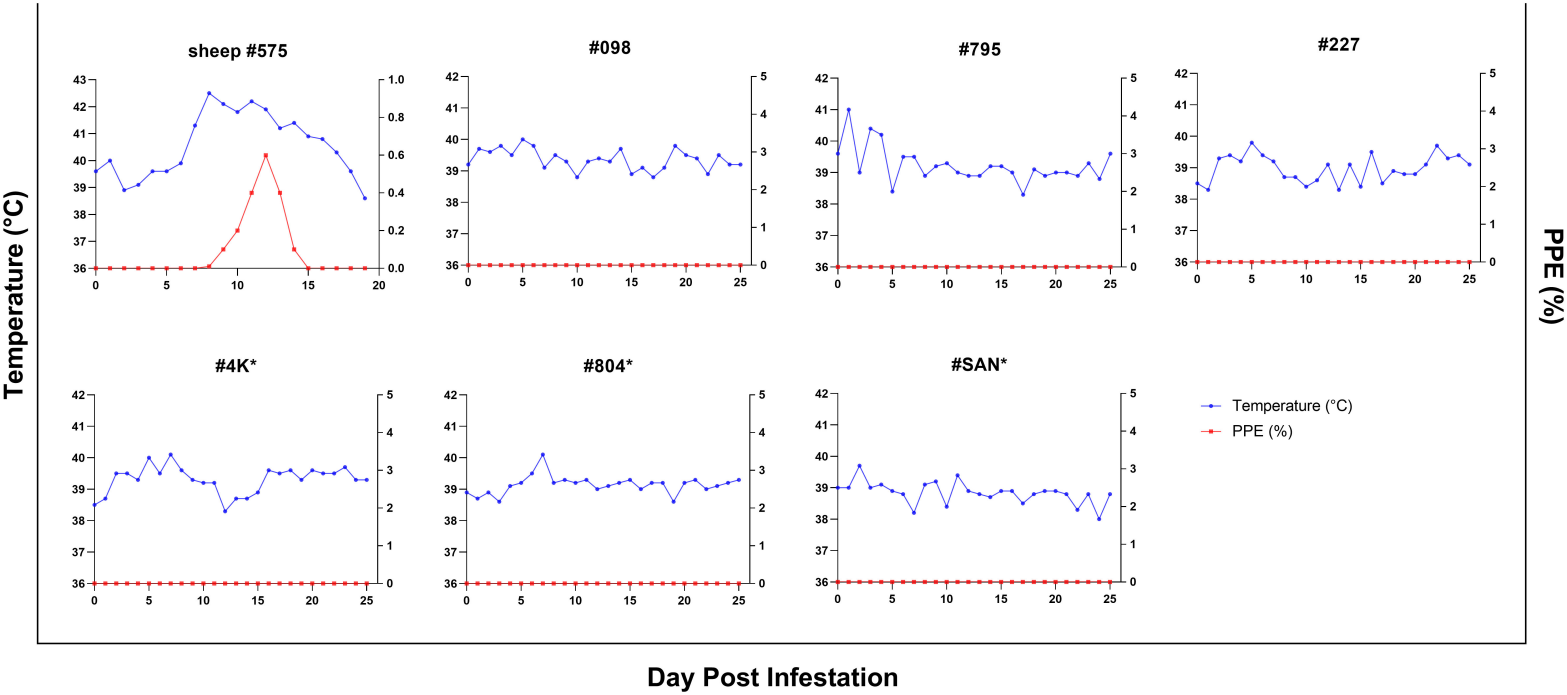
Parasitemia (PPE) and body temperature of goats in splenectomized (*) and spleen-intact groups infested with *R. bursa* ticks infected with *B. ovis*. #575 represents a spleen-intact control sheep treated in identical fashion.

In addition, experimental infection was conducted using *B. ovis*-infected ticks. For control purposes, sheep #575 was infested with 50 female and 50 male *B. ovis*-infected *R. bursa* ticks. On the seventh day following infestation, parasites were detected in the sheep peripheral blood. As the infection progressed, the sheep developed a fever, with temperatures peaking at 42.5°C on the 8^th^ day post-infestation. The maximum percentage of parasitemia (PPE) was recorded as 0.6% on the 12^th^ day, and the sheep succumbed to acute babesiosis on the 20^th^ day after infestation (**Figure 5**).

The same colony of infected ticks was used to infest splenectomized (#4K, #804, #SAN) and spleen-intact goats (#098, #795, #227), with each goat receiving 50 female and 50 male ticks. Goats were monitored for the presence of *B. ovis* by microscopy and nPCR, as in the experiment with infected blood described above, for 60 days after infestation. Clinical signs of babesiosis were not observed in any of the goats. However, *B. ovis* DNA was detected by nPCR in goat #098 starting on the 12^th^ day and continuing until the 54^th^ day post-infestation, in goat #795, *B. ovis* was detected by nPCR only on the 19^th^ day, while in goats #4K, #804, #SAN, and #227, *B. ovis* was not detected at all during the course of the experiment (**Figure 4**).

### *Babesia ovis*-infected goats are not a source for transstadial and transovarial parasite transmission by *R. bursa* ticks

To evaluate transstadial transmission, *Babesia* spp.-free *R. bursa* larvae were used to infest goats (#098, #795) that were found to be positive by nPCR. Engorged nymphs were collected from the goats 12-16 days post-infestation. The collected engorged nymphs were incubated (25 ± 1°C, 70 ± 10% RH) to obtain unfed adult ticks. The unfed adult ticks (50 females, 50 males) were then used to infest splenectomized sheep (#K2, #K3). The infested ticks were collected 8-10 days after feeding. All collected male and female ticks from #K2 and #K3 were individually placed into Eppendorf tubes, and after DNA extraction, examination using nPCR determined that they were negative for *B. ovis*. The splenectomized sheep were monitored for 60 days using both microscopic examination and nPCR, and both transmissions. Sheep #K2 and #K3 were negative for *B. ovis* throughout the observation period.

To assess transovarial transmission, engorged female ticks were collected from goats #098 and #795. Unfed offspring larvae were obtained from these ticks and used to infest rabbits (#Rabbit-1, #Rabbit-2). Fourteen to sixteen days after infestation, engorged nymphs were collected from the rabbits. These nymphs developed into unfed adult ticks, which were then used to infest splenectomized sheep (#K1, #K4). The ticks engorged within 8-10 days of feeding and were collected for analysis. As in the transstadial transmission experiment, both male and female ticks were examined by nPCR and found to be negative for *B. ovis*. The transmission sheep #K1 and #K4 were monitored for 60 days using both microscopic examination and nPCR, and both were determined to be negative for *B. ovis*.

## Discussion

The pathogenesis of babesiosis in goats remains under-researched, despite its potential to cause fatal infections in sheep. Goats and sheep usually co-exist on farms and there is the need to determine whether goats can act as reservoirs for the parasite in order to establish effective strategies for the control of *B. ovis*. Taking these factors into consideration, this study aimed to investigate the pathogenicity of *B. ovis* in splenectomized and spleen-intact goats. The study revealed significant insights into the host specificity and transmission dynamics of *B. ovis*. Notably, *B. ovis* did not cause overt clinical infection in goats, even after direct inoculation with infected blood or experimental infestation with *B. ovis*-infected ticks. Infections were observed in only two spleen-intact goats (#098, #795) infected via ticks, but these infections were subclinical and asymptomatic. This suggests goats may have species-specific immunity or resistance, as they showed no typical babesiosis symptoms like fever, anemia, and jaundice. More importantly, the species-specific resistance against this parasite occurred independently of the spleen and mode of infection. In contrast, intravenous administration of a *B. ovis* Alacakaya stabilate to sheep resulted in severe clinical symptoms and high parasitemia, showing high virulence. Additionally, experimental infestation with *B. ovis*-infected *R. bursa* ticks led to clinical infection in sheep, with detectable parasitemia, high fever, anemia, and eventual mortality. *Babesia ovis* was detected by nPCR in some goats but did not establish a sustained infection, indicating innate resistance. Our findings align with previous review papers suggesting that *B. ovis* presents sub- clinically in goats (Friedhoff, 1997; De Waal, 2000; Schnittger et al., 2022). However, a previous study has reported natural infection of *B. ovis* in goats, with PPE ranging from 0.1% to 1%, and canonical symptoms of babesiosis, such as fever, anemia, and icterus, in the infected animals. These studies, however, did not provide information on the duration of parasitemia, treatment, or recovery outcomes (Esmaeilnejad et al., 2012, 2020).

Research on *Babesia bovis*, the primary causative agent of bovine babesiosis, has shown high levels of genetic differentiation and diversity worldwide (Ozubek et al., 2020). Although population genetics studies on *B. ovis* are limited, a study reported high genetic diversity and differentiation in the *B. ovis* population in Turkiye (Mira et al., 2020). Different genetic strains of *B. ovis* may cause babesiosis in goats; however, the *B. ovis* Alacakaya strain used in our study did not induce any clinical infection in goats, despite being highly virulent for sheep. Additionally, different goat breeds may contribute to the development of clinical babesiosis. In this study, local breed hairy goats were used for experimental infection, but natural cases of babesiosis have been reported in Marghoz and Raeini goats in Iran (Esmaeilnejad et al., 2020). Another study found that while Chinese Tan sheep erythrocytes are susceptible to *Babesia* sp BQ1 (Lintan), French Vendéen sheep erythrocytes are not, indicating breed-specific susceptibility (Guan et al., 2010a). A similar study was conducted using virulent and attenuated strains of *B. bovis* to experimentally infect water buffaloes (Benitez et al., 2018). This experimental infection did not result in any clinical signs of babesiosis in the water buffaloes. During the experimental infection with the attenuated strain, *B. bovis* was detectable using nPCR, whereas it was not detectable with the virulent strain. As a result, it has been reported that buffaloes control babesiosis caused by *B. bovis* much more effectively than cattle (Benitez et al., 2018). Moreover, the experimental challenge of Nilgai antelope with a virulent *B. bovis* strain resulted in no signs of infection. Animals were nPCR-negative for the parasite and did not develop antibodies to *B. bovis*, further illustrating the variability in susceptibility among different species (Johnson et al., 2024). Collectively, these findings suggest that genetic and immunological factors intrinsic to each host species or breed play a crucial role in determining the outcome of *Babesia* infections.

In contrast to this study, experimental infections with *B. motasi*, *Babesia* sp. Lintan, and *B. aktasi* in goats yielded results, indicating varying degrees of clinical manifestations despite low to high parasitemia levels (Lewis et al., 1981; Guan et al., 2010b; Ozubek et al., 2023b). *Babesia motasi* infections caused mild anemia, moderate parasitemia, and mild fever in spleen-intact goats (Lewis et al., 1981). The varying clinical outcomes observed in goats infected with different species of *Babesia* highlight the complexity of host-parasite interactions. Understanding the factors governing these differences is essential for targeted interventions, such as vaccines or therapeutics, to combat these infections. Despite being a closely related species, goats exhibit notable resistance to tick-borne pathogens compared to sheep, leading to lower mortality rates. Investigating the innate and adaptive immunological responses and genetic factors conferring this resistance could provide novel avenues for livestock health management. Furthermore, goats' well-known resistance to diseases and their ability to thrive in harsh environments further support the findings of this study (Nair et al., 2021). Their robust immune system, characterized by a higher proportion of lymphocytes in circulation compared to neutrophils (Daramola and Adeloye, 2009), likely contributes to their resistance against tick-borne and other pathogens. Additionally, their resistance to experimental infections with the louping-ill virus (LIV) (Reid et al., 1984) and the toxic effects of oak ingestion further demonstrate their capacity to withstand various challenges (Smith and Sherman, 2009), as consistent with our data.

The data suggest that resistance to *B. ovis* in naïve goats is independent of spleen function and indicates the possible involvement of innate immune mechanisms in controlling the infection. This observation highlights the importance of considering innate immune responses, rather than adaptive mechanisms, as playing important roles in their resistance to *Babesia* infections (Torina et al., 2020; Bastos et al., 2022). Consistently, both spleen-intact and splenectomized goats, which had never been exposed to *B. ovis*, demonstrated resistance to infection in this study. This innate resistance may be the result of evolutionary adaptations that have led to unique immune responses in goats. The rapid activation of innate immune components, such as macrophages and neutrophils, along with the involvement of pattern recognition receptors (PRRs) like Toll-like receptors (TLRs) and nucleotide-binding oligomerization domain (NOD)-like receptors, could significantly contribute to the early and effective defense against *Babesia* infections (Torina et al., 2020). Understanding these mechanisms could provide valuable insights into developing control strategies for *Babesia* and other tick-borne diseases in livestock. Furthermore, the absence of infected erythrocytes in goats suggests a poor affinity of the parasite for the target erythrocytes. This raises the possibility of differences in erythrocyte surface characteristics and potential receptors between goats and sheep, which may limit the parasite's ability to invade. Conducting further experiments, such as culturing *B. ovis* in goat versus sheep erythrocytes, along with comparative analyses of erythrocyte surface molecules or distinct erythrocyte metabolism, could shed light on these observations. A better understanding of these mechanisms could ultimately be exploited to design more effective control measures against ovine babesiosis.

To investigate the epidemiological significance of goats in the transmission cycle of *B. ovis*, we conducted tick transmission experiments to determine whether goats could serve as reservoirs capable of transmitting *B. ovis* to *R. bursa* ticks. The rationale for these experiments is based on findings from other tick-borne protozoan infections, where both acute and chronic infections have been shown to contribute to vector transmission of certain *Babesia* and *Theileria* species (Yeruham et al., 2001; Gray et al., 2002; Howell et al., 2007; Ueti et al., 2012; Erster et al., 2016; Peckle et al., 2022). Previous research has shown that ticks feeding on animals with either acute or chronic infections can sometimes acquire and transmit *Theileria* and *Babesia* species. For instance, Howell et al. (2007) demonstrated that ticks feeding on persistently infected calves were capable of transmitting infection, though this ability depended on both the infection status and the timing of vector exposure. In some cases, larvae failed to transmit the pathogen from persistently infected animals, while in other instances, successful transmission occurred (Howell et al., 2007). Similarly, Gray et al. (2002) found that *Ixodes ricinus* ticks could be infected by *Babesia microti* HK and GI strains when feeding on acutely, but not chronically, infected gerbils (Gray et al., 2002). In some instances, ticks feeding on animals with chronic infections have been able to acquire and transmit the pathogen. For example, Peckle et al. (2022) showed that chronically infected horses successfully transmitted *Theileria equi* to *R. microplus* ticks (Peckle et al., 2022). Similarly, Ueti et al. (2012) reported that even horses treated with chemotherapeutic agents like imidocarb, which appeared healthy, still posed a transmission risk because subclinical carriers could continue to sustain the pathogen cycle within tick populations (Ueti et al., 2012). Based on these observations, we hypothesized that goats, which can harbor *B. ovis* and share environments with sheep, might contribute to the maintenance and transmission of the parasite to tick vectors. To test this, we designed a tick transmission experiment in which goats were infested with both larvae and adult *R. bursa* ticks, following methodologies similar to those used in transmission studies of other *Babesia* and *Theileria* species. Although limited in size and scope, our study involved infesting goats with both life stages of *R. bursa*. While molecular methods confirmed the presence of *B. ovis* in goats, we did not observe any transmission of the parasite to the ticks. This suggests that goats may not play a significant epidemiological role in the transmission of *B. ovis*. This outcome aligns with findings in the literature indicating that sufficient parasitemia is crucial for pathogen transmission. Studies on *Babesia* species have shown that the level of parasitemia in host animals directly impacts the ability of ticks to acquire the pathogen (Yeruham et al., 2001; Gray et al., 2002; Howell et al., 2007; Firat et al., 2024). In our study, the absence of detectable transmission may be due to low or transient parasitemia in goats, preventing ticks from acquiring *B. ovis* during feeding. These results suggest that, while acute and chronic infections of *Babesia* and *Theileria* species can sometimes lead to successful pathogen transmission, goats may not achieve the parasitemia threshold necessary for effective transmission of *B. ovis* to ticks. This finding has significant implications for understanding the role of different hosts in the transmission dynamics of tick-borne pathogens. It supports the focus on sheep as the primary reservoir in *B. ovis* control strategies and suggests that goats may have limited epidemiological importance in the transmission cycle of *B. ovis*. Further studies are needed to confirm this and clarify the potential role of goats in *B. ovis* epidemiology.

In conclusion, several lines of evidence in this study suggest that goats are not suitable hosts for *B. ovis*, as they exhibit resistance to clinical disease and do not support tick-borne transmission. Further research into the immunological responses of goats to *Babesia* infections could provide valuable insights into the mechanisms underlying host resistance or susceptibility. Additionally, assessing the economic impact of babesiosis in goats would be crucial for implementing effective control measures and mitigating losses in affected regions. Therefore, it is essential to consider ovine and caprine babesiosis separately due to their distinct pathogenicity profiles and host responses. It is likely that the factors contributing to this resistance may be related to innate immunity mechanisms rather than adaptive immunity. Understanding these innate immune responses in goats could pave the way for developing new strategies to control babesiosis in other susceptible species. Further studies in this area are warranted to fully elucidate the underlying mechanisms and to develop targeted interventions. Moreover, future studies should also focus on examining "heterologous" goats, from different breeds or from regions that are not endemic to *B. ovis*. This could help determine if the observed resistance is consistent across various goat populations and provide a broader understanding of the factors contributing to host resistance. In addition, it is crucial to conduct additional transmission studies to explore the role of goats in the epidemiology of *B. ovis*, in order to determine whether they effectively contribute to the parasite's life cycle under specific conditions. Such comprehensive research will be instrumental in enhancing our knowledge of babesiosis and improving control strategies in diverse geographic and ecological settings.

## Data Availability

The raw data supporting the conclusions of this article will be made available by the authors, without undue reservation.
